# The effect of magnocellular-based visual-motor intervention on Chinese children with developmental dyslexia

**DOI:** 10.3389/fpsyg.2015.01529

**Published:** 2015-10-06

**Authors:** Yi Qian, Hong-Yan Bi

**Affiliations:** ^1^Key Laboratory of Behavioral Science, Institute of Psychology, Chinese Academy of Sciences, BeijingChina; ^2^University of Chinese Academy of Sciences, BeijingChina

**Keywords:** developmental dyslexia, magnocellular pathway, coherent motion detection, visual-motor intervention, Chinese reading

## Abstract

Magnocellular (M) deficit theory points out that the core deficit of developmental dyslexia (DD) is the impairment in M pathway, which has been evidenced in many previous studies. Based on the M deficit, some researchers found that visual intervention focusing on M deficit improved dyslexics’ M function as well as reading abilities. However, the number and reliability of these training studies were limited. Therefore, the present study conducted an M-based visual-motor intervention on Chinese children with DD to investigate the relationship between M deficit and Chinese DD. Intervention programs included coherent motion detection, visual search, visual tracking, and juggling, which were related to M function. The results showed that M function and phonological awareness of training dyslexic group were improved to a normal level as age-matched normal children after intervention, while non-training dyslexics did not. It supported M deficit theory, and suggested M deficit might be the core deficit of Chinese DD.

## Introduction

Developmental dyslexia (DD) is a neurobiological reading disorder. Individuals with DD have difficulties in accurate or fluent word recognition, spelling, and word decoding despite adequate instruction and intelligence (Lyon et al., 2003). Although researchers have made efforts in studying on DD, the cause of DD remains controversial. Some researchers pointed out that the cause of DD could be traced back to a more general perceptual dysfunction. Magnocellular (M) deficit theory postulates that the core deficit of DD is the impairment in M pathway (Stein, 2001). M pathway starts from retinal M ganglion cells in retina, then visual information is conveyed to the M layers of the dorsal lateral geniculate nucleus (LGN) of the thalamus. Then, information was project to the primary visual cortex, and further transferred to the posterior parietal cortex (PPC) via the dorsal stream (also known as the “where” stream), which has been implicated in object localization, motion perception, visual attention, and goal-directed behavior. The dorsal stream includes middle temporal (MT) area, which is known to play a key role in motion perception and is specifically activated when observers are presented with random dot kinematograms (RDKs) containing coherent motion (CM; Goodale and Westwood, 2004; Boden and Giaschi, 2007).

Compared with chronological age-matched (CA) controls, individuals with DD showed less sensitivity in detecting CM (Cornelissen et al., 1995; Talcott et al., 1998, 2003; Witton et al., 1998; Hansen et al., 2001; Conlon et al., 2004; Pellicano and Gibson, 2008). Moreover, CM sensitivity of DD was significantly correlated with pseudoword reading, which reflected the phonological processing skills (Talcott et al., 1998; Witton et al., 1998). Pre-reading children at familial risk of DD exhibited the disability in detecting CM, suggesting deficits in M pathway occurred before reading commencement (Kevan and Pammer, 2008). A meta-analysis study obtained a relatively large effect size of CM sensitivity in comparison between CA controls and DD, confirming the reliability of CM deficits of DD (Benassi et al., 2010). However, some studies did not support M deficit theory. Ramus et al. (2003) found that only 2 of 16 dyslexic adults had visual M deficit. Meanwhile, these two visually impaired dyslexics also had auditory and phonological problems, suggesting that visual M deficit was not an independent core deficit of DD. Sperling et al. (2005) pointed out that deficits in noise exclusion, not M processing, contributed to the etiology of dyslexia. Additionally, Skottun (2015) pointed out that CM deficit should be attributed to deficits of dorsal stream rather than M pathway; namely, M pathway and dorsal stream should be distinguished. Although M deficit theory is still debatable, M deficits have been extensively evidenced in DD.

Some researchers tried to conduct intervention studies on DD. Solan et al. (2004) adopted perceptual accuracy, visual efficiency, guided reading, visual search, and visual scan as training programs. After 15 training sessions, disabled readers’ CM sensitivity, pseudoword reading skills, and reading comprehension were improved (Solan et al., 2004). However, the enhancement of M function and reading skills might result from the print reading training rather than visual training, because the guided reading program used words as stimuli. Lawton (2011) conducted a direction discrimination task on DD twice a week for 14 weeks, and found that dyslexics enhanced contrast sensitivity (an index of M function) as well as a mass of reading skills, including reading rates, spelling, word identification and comprehension. The relatively small sample size (only three dyslexics) might limit the reliability of training effects. In a cohort of normal adult readers, either CM or parallel line detection (parvocellular task) training increased the speed of lexical decision, but the correlation between accuracy improvement in lexical decision and improvement of visual function was only found in the CM training group (Chouake et al., 2012). The result suggested that M training seemed to have an advantage over general visual training on reading performance [reaction time (RT) as well as accuracy]. These studies consistently showed that M-based intervention ameliorated reading skills, suggesting the causal role of M pathway in reading development. However, a recent functional magnetic resonance imaging (fMRI) study on dyslexia did not support this opinion. Although dyslexic children showed lower MT activity than CA controls, but similar activity to reading level-matched controls. What’s more, the researchers conducted an 8-week phonological-based reading training on DD. They found that dyslexics’ MT activity along with reading skills [i.e., real word and pseudoword reading skills and phonological awareness (PA)] were improved, while their performance did not change in the control period (in which a math intervention or no intervention was carried out; Olulade et al., 2013). The result suggested the M deficits attributed to lower reading level and less reading experience rather than the cause of DD.

The above studies were conducted in alphabetic languages. In contrast to the writing systems with specific grapheme-to-phoneme correspondence, Chinese is a logographic writing system. Chinese characters have complex visual structures. So, visual processing skills are important for Chinese reading development (Chung et al., 2008; Li et al., 2012; Yang et al., 2013). Previous studies found that Chinese dyslexic children had higher CM thresholds than CA controls (Meng et al., 2011; Qian and Bi, 2014), suggesting that Chinese DD had impairments in visual M pathway. An event-related potential (ERP) study showed Chinese DD induced smaller amplitude of visual mismatch negativities (vMMNs) than both age-matched and reading level matched children in the visual M condition (moving gratings; Wang et al., 2010). A recent intervention study found visual perceptual training significantly decreased the discrimination threshold of visual texture discrimination task (TDT) for Chinese DD. Meanwhile, DD who accepted training exhibited significant and long-lasting enhancement in reading fluency (Meng et al., 2014). However, this study focused on training of general visual perception. Then, how about M pathway function?

Based on previous evidence of M deficits in Chinese DD (Meng et al., 2011; Qian and Bi, 2014), the present study examined whether M-based visual-motor intervention had a positive effect in M function and relevant reading-related cognitive skills of DD. Dyslexic children accepted 10 sessions of visual-motor training focusing on M pathway. Meanwhile, CM detection and reading-related skills were tested on training DD, non-training DD and CA groups. According to previous studies in both alphabetic languages and Chinese, DD exhibited deficits in PA and rapid naming (RAN), which played important roles in reading development (McBride-Chang and Manis, 1996; Wolf and Bowers, 1999; Ho et al., 2000, 2002). Moreover, the two skills were closely associated with M function (Hulslander et al., 2004; Kinsey et al., 2004; Ben-Shachar et al., 2007). Therefore, we adopted PA test and digit RAN test as indexes of children’s reading skills.

## Materials and Methods

### Participants

The participants were recruited from ordinary primary schools in Beijing. The study was conducted under the informed consent of their parents, and was approved by the Institutional Review Board of the Institute of Psychology, Chinese Academy of Sciences. All of the participants were right-handed, and had normal hearing and normal or corrected-to-normal vision without ophthalmological or neurological abnormalities. The inclusionary criteria for DD were consistent with previous studies in mainland China (Shu et al., 2006; Wang et al., 2010; Meng et al., 2011; Zhao et al., 2014), including the IQ was above 85 as measured by Combined Raven’s Test (Li et al., 1989), meanwhile the written vocabulary test score was at least one and a half standard deviations below corresponding age norm in the Standard Character Recognition Test (Wang and Tao, 1996). Seventeen children with DD and 11 CA children (three female, mean age: 10.42 years, and range: 9–11 years) took part in this study. The children with DD were randomly divided into two groups. One DD group accepted M-based visual-motor intervention (eight children, two females, mean age: 10.63 years, and range: 9–11 years), while another group did not (nine children, two females, mean age: 10.11 years, and range: 9–11 years). Characteristics of participants were shown in **Table 1**.

**Table 1:** Characteristics of two developmental dyslexia (DD) groups and one chronological age-matched (CA) group.

	Training DD 1 (*n* = 8)	Non-training DD 2 (*n* = 9)	CA 3 (*n* = 11)	*F*	Group comparison
Age (years)	10.63 (0.52)	10.11 (0.33)	10.42 (1.07)	1.10	➀ = ➁ = ➂
IQ	100.13 (11.37)	105.63 (10.35)	109.82 (11.91)	1.70	➀ = ➁ = ➂
Vocabulary	2111.02 (306.91)	2067.30 (502.78)	2807.48 (349.70)	11.10^∗∗∗^	➀ = ➁ < ➂

Standard deviations were shown in the parentheses; ***p < 0.001.

### Reading-related Tests

#### PA test

An oddball paradigm (Bradley and Bryant, 1978) was adopted. Within a trial, three characters were presented orally by the experimenter, and participants were asked to pick out a phonologically odd item among them. There were three types of oddity: onset, rime, and lexical tone. For example, for the three items “tan4”, “tong3”, and “ji1”, “tan4” and“tong3” had the same onset “t”, which was different from “ji1”. Meanwhile, the three items were completely different in rime and lexical tone in order to control for possible confounds. A total of ten trails for each type of oddity was presented. The accuracy of responses was recorded. The test-retest reliability was 0.77.

#### RAN Test

Five digits (2, 4, 6, 7, and 9) were used. Digits were repeatedly presented visually in random order on a 6 × 5 row–column grid. Participants were asked to name each digit in sequence as quickly as possible. The test was conducted twice, and the scores were averaged as the final score. The total time (s) taken to name all digits was collected and converted to a per-second score. The test–retest reliability was 0.89.

### Magnocelluar Function Test

We adopted CM detection task to measure magnocelluar pathway function. The experimental paradigm was similar to what used in the study of Solan et al. (2004). Two patches of 300 randomly moving white dots with a speed of 7°/s and a lifetime of 225 ms were presented on the left and right sides of screen with dark background. The luminance of dots was 125 cd/m^2^, and the luminance of background was 0.39 cd/m^2^, Michelson contrast was 99.4%. The two patches were separated by a horizontal distance subtending 5°, and the size was 10° wide and 14°high. The patches of dots were presented for 2300 ms in each trial. In one patch, all dots moved randomly, while the other patch had a certain percentage of dots moving coherently leftward or rightward. Participants had to judge which patch had such coherently moving dots after patches disappeared. CM threshold was obtained according to a 1-up–1-down staircase procedure; incorrect responses led to an increase in the number of coherent moving dots by 1%, while correct responses led to a decrease by 1%. After 10 reversals, a session was terminated. Threshold was defined by the mean of the number of coherent moving dots of the last six reversals. The experiment included three sessions, and the final CM threshold was the mean of them. The test–retest reliability was 0.74.

### Magnocellular (M)-based Visual-motor Intervention

**FIGURE 1:**
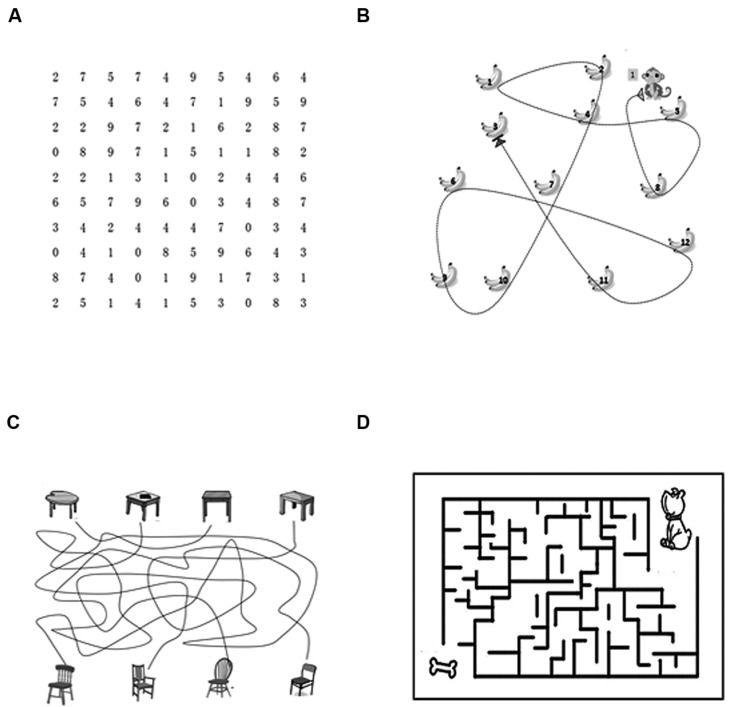
**The examples of visual search and visual tracking tasks. (A)** The example of visual search; **(B)** The example of dynamic tracking; **(C)** The example of line puzzle (a task of static tracking); **(D)** The example of maze puzzle (a task of static tracking).

The present study adopted M-based visual-motor intervention programs to train dyslexics’ M function. One training session included three training projects:

(1) *CM detection task*. This task aimed to train M function directly. The parameters were similar to that in the above M function test.
(2) *Visual search and visual tracking tasks*. The tasks focused on eye movement, object localization and visual spatial attention, which were the function of M pathway (Boden and Giaschi, 2007). These tasks were also involved in previous intervention studies (Solan et al., 2004; Lawton, 2011). (i) In the visual search task, participants were asked to search certain digits (0–9) rapidly and delete them in the sequence from small to large (i.e., 0, 1, 2, 3 ……9) on a paper of 100 digits. (ii) Visual tracking task included dynamic tracking and static tracking. In dynamic tracking task (12 trials), participants were required to gaze the moving object, pursuit its moving direction, and localize its final position. Static tracking task involved line puzzle (six trials) and maze puzzle (six trials). In the line puzzle task, participants were asked to follow the correct line to find the correct object in connection with the target; in the maze puzzle task, participants were required to find the correct path from entrance to exit. The paradigms of these tasks referred to a previous study (Yu et al., 2006) and the Internet (http://www.eyecanlearn.com/). Stimuli in visual search and static visual tracking were presented on papers, while stimuli in dynamic tracking were presented by the computer. The stimuli were different in each training session, and the examples were shown in **Figure 1**.

(3) *Juggling*. An fMRI study (Draganski et al., 2004) found that after 3-month juggling training, participants demonstrated a significant transient bilateral expansion in grey matter in MT area. This study suggested that juggling was associated with visual perception and spatial anticipation of moving objects, and was a stronger stimulus for plasticity in the visual areas than in the motor areas (Draganski et al., 2004). Therefore, juggling was regarded as a M-based intervention program, and adopted to induce the changes of MT area.

### Intervention Procedure

The training DD group accepted ten sessions of M-based visual-motor intervention within 5 weeks with two sessions per week. One session cost about one hour. When training group conducted training programs, non-training dyslexic group, and CA group did free activities. All the three groups accepted reading-related tests and M function test before and after the intervention course.

## Results

CM thresholds, accuracy in PA test, digit RAN speed before and after training for three groups were shown in **Table 2**. Repeated measures ANOVA were conducted to analyze CM thresholds, accuracy of PA and RAN speed, with group (training DD and non-training DD) as a between-subject factor, and time (pre- and post-) as a within-subject factor. Additionally, we compared the performance difference of the two DD groups and CA group in the three tests, so as to explore dyslexics’ deficits before and after training when compared with normal readers.

**Table 2:** The performance of pre-test and post-test for three groups.

	Pre-			Post-		
	Training DD	Non-training DD	CA	Training DD	Non-training DD	CA
CM	75.88 (26.13)	83.00 (24.61)	46.41 (19.59)	34.19 (11.31)	79.02 (33.43)	49.80 (30.13)
PA	0.47 (0.12)	0.53 (0.15)	0.72 (0.15)	0.63 (0.16)	0.54 (0.16)	0.71 (0.14)
RAN	2.66 (0.42)	2.69 (0.42)	3.19 (0.56)	2.87 (0.37)	2.85 (0.51)	3.50 (0.71)

*Standard deviations were shown in the parentheses. CM, coherent motion threshold; PA, accuracy in phonological awareness test; RAN, digit rapid naming speed (number per second).*

For CM thresholds, the main effects of group (training DD group and non-training DD group) and time were significant [*F*(1,15) = 6.36, *p* < 0.05; *F*(1,15) = 11.07, *p* < 0.01], the interaction between group and time was also significant [*F*(1,15) = 7.55, *p* < 0.05]. Further simple effect analysis showed that there was no significant difference between the two DD groups in the pre-test, while dyslexics in training group had a significantly lower threshold than those in non-training group (*p* < 0.01) in the post-test. For training DD group, post-threshold was significantly lower than pre-threshold (*p* < 0.01), but there was no significant difference for non-training group.

With respect to PA, the main effects of time was significant [*F*(1,15) = 20.64, *p* < 0.001], while the main effects of group (training DD group and non-training DD group) was not [*F*(1,15) = 0.04, *p* > 0.05]. The interaction between them was significant [*F*(1,15) = 14.32, *p* < 0.01]. Simple effect analysis showed that group differences were not significantly different in both pre-PA test and post-PA test The difference of accuracy between pre- and post-test was significant for training DD group (post-accuracy was higher; *p* < 0.001), but not for non-training DD group.

For digit naming speed, the main effect of time was significant [*F*(1,15) = 8.23, *p* < 0.05], post-naming was faster than pre-naming. The main effect of group and the interaction between group and time were not significant.

In order to further explore whether the intervention could improve dyslexics’ performance to a normal level, one-way ANOVA was conducted to compare the performance of two DD groups with that of CA group before and after intervention. The results showed that the differences of three groups were significant in pre- and post- CM, PA, and RAN tests; pre-CM: *F*(2,25) = 7.05, *p* < 0.01; pre-PA: *F*(2,25) = 8.20, *p* < 0.01; pre-RAN: *F*(2,25) = 3.88, *p* < 0.05; post-CM: *F*(2,25) = 5.92, *p* < 0.01; post-PA: *F*(2,25) = 3.17, *p* = 0.06; and post-RAN: *F*(2,25) = 4.31, *p* < 0.05. *Post hoc* analyses indicated that, in the pre-tests, two DD groups had significantly higher CM thresholds (*p*s < 0.05), lower accuracy in PA test (*p*s < 0.01), and slower RAN (*p*s < 0.05) than CA group, while there was no significant difference between the two DD groups in each of three tests. For post-CM, training DD group and CA group had lower thresholds than non-training group (*p*s < 0.05), and there was no difference between training DD group and CA group. For post-PA, only CA group had significantly higher accuracy than non-training group (*p* < 0.05), while there was no significant difference between CA and training groups and between training and non-training groups. For post-RAN, CA group had faster naming speed than two DD groups (*p*s < 0.05), while there was no significant difference between DD groups.

## Discussion

The present study investigated whether M-based visual-motor intervention could improve M function and reading-related cognitive skills. Compared with age-matched normal children, children with DD exhibited deficits in CM detection, PA and RAN in pre-tests. After intervention, training DD group decreased CM thresholds (i.e., increased CM sensitivity) and increased PA, while non-training DD did not. Additionally, the performance of training DD group in post-CM and PA tests was equal to that of CA group.

Magnocellular-based visual-motor training improved the CM sensitivity of children with DD to a normal level. However, there was no significant difference between pre- and post-CM thresholds for non-training dyslexics. Consistent with previous alphabetic studies (Solan et al., 2004; Lawton, 2011), the present result suggested the M pathway function of dyslexic children benefited from visual intervention. However, the intervention programs included direct CM tasks, then was the enhancement just a practice effect? Actually, dyslexics’ deficits in CM were relatively persistent, and not influenced by practice (Conlon et al., 2009), so the enhancement here might not be merely a practice effect, and might benefit from other training tasks. The current visual-motor intervention involved training focusing on visual search and tracking, which might be related with oculomotor control and visual spatial attention (Hooge and Erkelens, 1999; Kramer et al., 1999; de Brouwer et al., 2002). Juggling has been evidenced induced structural changes in MT area (Draganski et al., 2004). Because of the close association between structure and function (Bullmore and Sporns, 2009), juggling might also exert a positive effect on neural activities in MT areas and further directly influence motion perception. Therefore, dyslexics’ M function could be effectively ameliorated by an integrated M-based visual-motor intervention with diverse training programs. However, it was unknown which of these tasks played a dominant role in the improvement of M function, which should be further investigated in the future.

In the post-test, PA of dyslexic children with training was significantly improved, and the accuracy was equivalent to that of CA. However, the accuracy of non-training dyslexics did not differ in the pre- and post-tests, and was still lower than CA, which suggested that M-based visual-motor intervention improved dyslexics’ PA, supporting the M deficit theory. Many prior studies have found the association between M function and PA (Talcott et al., 1998; Witton et al., 1998; Hulslander et al., 2004; Kinsey et al., 2004; Ben-Shachar et al., 2007). Another training study found that after a 8-week phonological-based reading intervention, MT activity increased along with enhancement of PA (Olulade et al., 2013). It was implied that M deficit was the consequence of impoverished reading rather than the cause of DD. Whereas, Olulade et al. (2013) adopted “seeing stars” as the phonological-based intervention, which addressed visualization of letters, syllables, multisyllables, and words as well as motor/tactile and articulatory aspects of word presentation (Bell, 1997), thereby promoting visual imagery of orthographic presentations as well as PA. So, the intervention program might likewise influence visual system, consequently improving activation in MT area. In contrast, the intervention in the current study was pure perceptual training without the confusion of prints or phonological processing. Thus, it adequately supported M theory, and evidenced that M deficit might be the cause of DD.

Previous studies manifested an association between M function and RAN (Hulslander et al., 2004; Meng et al., 2011; Qian et al., 2015). However, all the three groups in the present study increased RAN speed in the post-test, suggesting the enhancement of RAN might be the outcome of natural development rather than a training effect. The training time was short in the present, which might lead to the lack of training effect on RAN. Therefore, it needed further study to explore whether more training sessions would induce larger improvement of naming speed for training dyslexics.

However, the current study had some limitations. Firstly, the relatively small sample size restricted the reliability of the current training effect. Secondly, the non-training groups did free activities, which were not manipulated. Thirdly, the current study lacked character reading or spelling tests, so it was unclear whether the enhancement of reading-related skills could be transferred to general character reading. Finally, all of the dyslexics here had deficits in M pathway. Thus, it remained unknown whether M-based intervention was beneficial to other subtypes of DD. Due to these limitations, caution was needed in drawing conclusions of intervention effect of M pathway. Further studies were needed to confirm the current result by enlarging sample size, manipulating control training programs, adding general character reading tests, and involving more subtypes of DD, etc.

## Summary

The present preliminary study showed M-based visual-motor training might improve dyslexics’ M function and PA, suggesting M-based intervention might be beneficial to Chinese DD.

## Conflict of Interest Statement

The authors declare that the research was conducted in the absence of any commercial or financial relationships that could be construed as a potential conflict of interest.
